# Differential modulation of AMPK/PPARα/UCP2 axis in relation to hypertension and aging in the brain, kidneys and heart of two closely related spontaneously hypertensive rat strains

**DOI:** 10.18632/oncotarget.4033

**Published:** 2015-05-22

**Authors:** Speranza Rubattu, Franca Bianchi, Carla Letizia Busceti, Maria Cotugno, Rosita Stanzione, Simona Marchitti, Sara Di Castro, Michele Madonna, Ferdinando Nicoletti, Massimo Volpe

**Affiliations:** ^1^ Department of Clinical and Molecular Medicine, School of Medicine and Psychology, Sapienza University of Rome, Ospedale S. Andrea, Rome; ^2^ Istituto di Ricovero e Cura a Carattere Scientifico (I.R.C.C.S.) Neuromed, Località Camerelle, Pozzilli, Italy; ^3^ Department of Physiology and Pharmacology, Sapienza University of Rome, Rome, Italy

**Keywords:** uncoupling protein 2, SHRSP, hypertension, target organ damage, aging

## Abstract

**Objectives:**

We examined expression protein of AMPK/SIRT1/PGC1α/PhoxO3a/PPARα/UCP2 pathway in brain, kidneys and heart of stroke-prone spontaneously hypertensive rat (SHRSP) vs stroke-resistant SHR (SHRSR) at different weeks of age, up to one year, in order to test the hypothesis that abnormalities within this pathway could associate with higher susceptibility of SHRSP to develop hypertension-related vascular damage.

**Background:**

SHRSP develops severe hypertension and related target organ damage. Marked reduction of uncoupling protein 2 (UCP2) expression upon high salt-low potassium diet associates with increased renal injury in SHRSP. UCP2 may represent a key mitochondrial protein involved in cardiovascular damage.

**Results:**

At 2 months of age a significant down-regulation of UCP2 expression at both mRNA and protein levels was found, along with reduced protein expression of all components of UCP2 regulatory pathway, in tissues of SHRSP but not of SHRSR, that progressed with hypertension development and aging. A significant increase of both oxidative stress and inflammation was detected in tissues of SHRSP as a function of age. SBP levels were significantly higher in SHRSP than SHRSR at 3 months of age and thereafter. At one year of age, higher degree of renal damage, with proteinuria and severe glomerular and tubulo-interstitial fibrosis, of cerebral damage, with significant vessel extravasation and stroke occurrence, and of myocardial damage was detected in SHRSP than SHRSR.

**Conclusions:**

The early significant reduced expression of the antioxidant AMPK/PPARα/UCP2 pathway that progressed throughout lifetime may contribute to explain higher predisposition of SHRSP to oxidative stress dependent target organ damage in the context of severe hypertension.

## INTRODUCTION

Uncoupling protein 2 (UCP2) is a inner mitochondrial membrane protein and a downstream effector of the AMPK/SIRT1/PPARα pathway. It plays a protective physiological role in several tissues by regulating fatty acid oxidation, mitochondrial biogenesis, substrate utilization and elimination of reactive oxygen species (ROS) [1]. Down-regulation of UCP2 is associated with increased oxidative stress, atherosclerosis, impaired vascular function and shorter lifespan in mice [2]. In contrast, UCP2 overexpression inhibits ROS production in vascular endothelial cells and preserves endothelial function by reducing ROS production and increasing nitric oxide bioavailability in obese diabetic mice [3]. UCP2 also show vascular beneficial effects in hypertension [4]. Taken together, these findings suggest that UCP2 is a key mitochondrial protein involved in protection from vascular damage. Interestingly, for all above mentioned functional properties, UCP2 may also connect longevity with metabolism, consistent with the “uncoupling-to-survive” hypothesis proposed by Brand [5].

The stroke-prone spontaneously hypertensive rat (SHRSP) develops severe hypertension and, as opposed to its related control strain, the stroke-resistant SHR, shows an increased occurrence of cerebrovascular accidents at older ages, with signs of cortical and subcortical infarcts and hemorrhages [6, 7]. Stroke is accelerated by exposure to a high sodium-low potassium diet (Japanese style diet), being always anticipated by renal damage [8, 9]. Notably, the gene encoding UCP2 maps nearby the lod score peak of a Quantitative Trait Locus for stroke (STR1) in SHRSP [10]. We have previously shown that UCP2 and proteins that lie upstream in the UCP2 regulatory pathway are down-regulated in kidneys of Japanese diet fed SHRSP but not in kidneys of SHRSR. This evidence suggests that changes in UCP2 expression contribute to higher predisposition of high salt fed SHRSP to renal damage [11]. Accordingly, a Brassica sprouts extract protects high salt fed SHRSP from renal damage through UCP2 upregulation [12].

Whether changes in UCP2 expression are associated with development of hypertension and aging and may contribute to the increased incidence of stroke and of other organ damage in SHRSP is currently unknown. We performed the present study to test the hypothesis that expression of UCP2 and of its upstream regulatory pathway could be differently modulated in relation to hypertension development and aging in the brain, as well as in the kidneys and heart, of SHRSP as compared to its related control strain.

## RESULTS

### Body weight, systolic blood pressure and proteinuria level

Table 1 shows body weight (BW), systolic blood pressure (SBP), and proteinuria levels at different times of life in both rat strains. At 2 months of age (8 weeks) both strains had developed hypertension, with a subsequent steeper rise of SBP values over the following months in the SHRSP as compared to SHRSR. The difference became significant at 3 months of age.

**Table 1:** Body weight, blood pressure and proteinuria levels in SHRSP and SHRSR at different weeks of age

SHRSR	6 weeks	8 weeks	10 weeks	12 weeks	24 weeks	one year
BW (g)	110 ± 1	158 ± 8^Σ^	194 ± 3^Φ^	275 ± 5^Φ^	321 ± 1^Φ^	357 ± 12^Φ^
SBP (mmHg)	108 ± 2	173 ± 5^Φ^	172 ± 1^Φ^	175 ± 2^Φ^	183 ± 6^Φ^	196 ± 2^Φ^
Proteinuria (mg/24hr)	1.6 ± 0.1	13 ± 2	28 ± 8	63 ± 2^Σ^	73 ± 3^Σ^	75 ± 10^ς^

SHRSR	6 weeks	8 weeks	10 weeks	12 weeks	24 weeks	one year
BW (g)	100 ± 2	148 ± 1^ω^	181 ± 6^#^	207 ± 14^♦^^#^	278 ± 10^**^^#^	299 ± 14^♦^^#^
SBP (mmHg)	108 ± 1	174 ± 4^#^	179 ± 2^#^	185 ± 2^*^^#^	193 ± 6^♦^^#^	207 ± 14^♦^^#^
Proteinuria (mg/24hr)	1.6 ± 0.01	18 ± 1.5^#^	63 ± 3^#^	104 ± 3^*^^#^	175 ± 24^♦^^#^	409 ± 32^♦^^#^

* *p* < 0.05, SHRSP vs corresponding time of life of SHRSR

** *p* < 0.01, SHRSP vs corresponding time of life of SHRSR

♦ *p* < 0.0001, SHRSP vs corresponding time of life of SHRSR.

Σ *p* < 0.02, SHRSR at each indicated time of life vs its own 6 weeks of age

ς *p* < 0.01, SHRSR at one year vs its own 6 weeks of age

Φ *p* < 0.0001, SHRSR at each indicated time of life vs its own 6 weeks of age

ω *p* < 0.02, SHRSP at 8 weeks vs its own 6 weeks of age

# *p* < 0.0001, SHRSP at each indicated time of life vs its own 6 weeks of age.

BW increased significantly with aging in both strains, although the rise was of significantly lower degree in SHRSP as compared to SHRSR starting at 3 months of age (Table 1).

Level of 24 hrs urinary protein excretion increased dramatically in SHRSP starting at 10 weeks of age and reached the highest values at one year of age. In contrast, urinary protein excretion increased only moderately, reaching a plateau at 3 months of age, in SHRSR. Differences between the two strains became significant at 3 months of age.

### Analysis of protein expression in brain, kidneys and heart from SHRSP vs SHRSR

**Figure 1:**
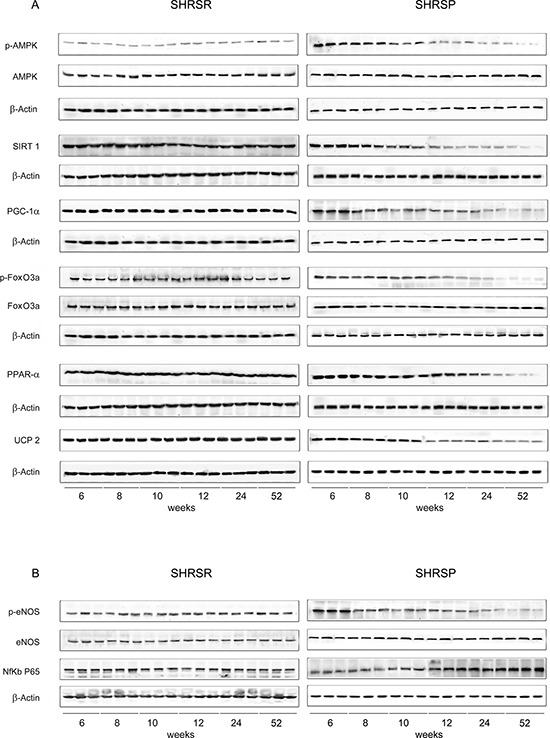
Panel A: western blots of AMPK/SIRT1/PGC1α/PhoxO3a/PPARα/UCP2 pathway throughout lifetime in the brains of SHRSR and SHRSP **Panel B:** western blots of phosphoeNOS/eNOS and of NfKb in the brains of the two strains.

**Figure 2:**
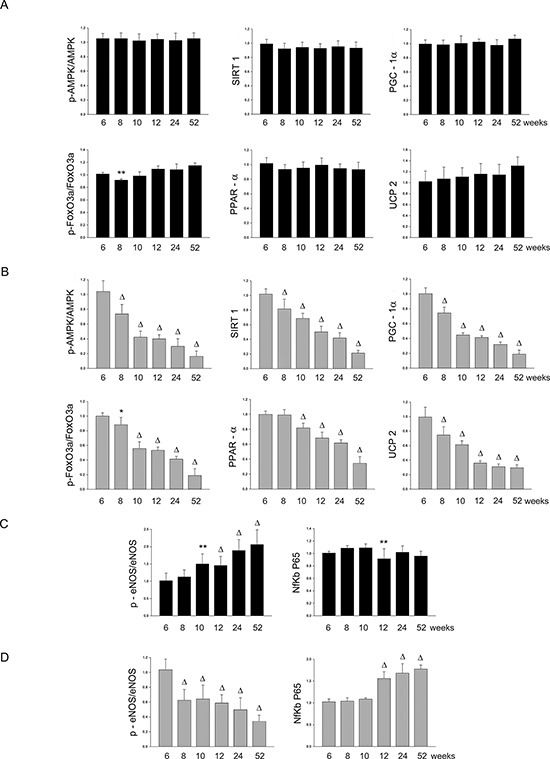
Panel A: densitometric analysis of each component of the AMPK/SIRT1/PGC1α/PhoxO3a/PPARα/UCP2 pathway at different weeks of age in the brains of SHRSR **Panel B:** same analysis in SHRSP; **Panel C:** densitometric analysis of phosphoeNOS/eNOS and of NfKb in SHRSR; **Panel D:** same analysis in SHRSP. **p < 0.01 vs 6 weeks of age; Δ*p* < 0.0001 vs 6 weeks of age

**Figure 3:**
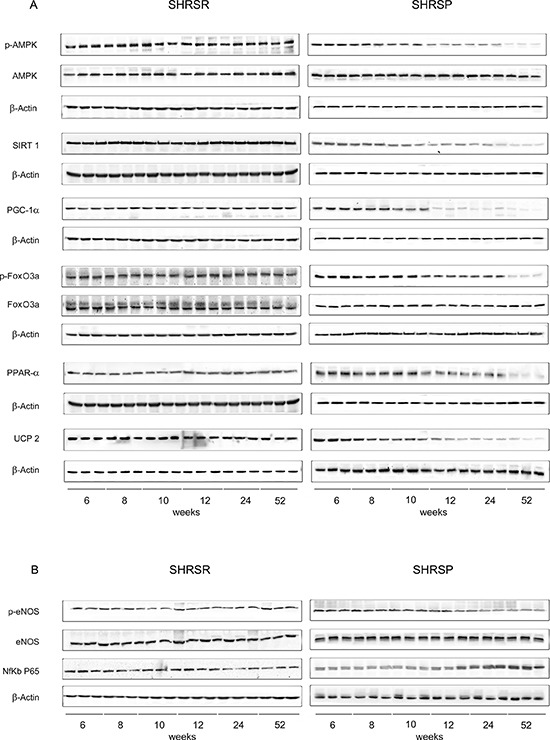
Panel A: western blots of AMPK/SIRT1/PGC1α/PhoxO3a/PPARα/UCP2 pathway throughout lifetime in the kidneys of SHRSR and SHRSP **Panel B:** western blots of phosphoeNOS/eNOS and of NfKb in the kidneys of the two strains.

**Figure 4:**
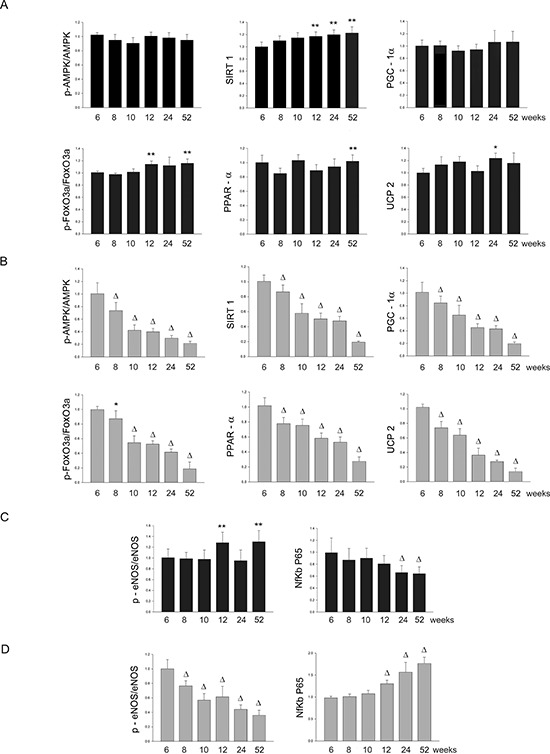
Panel A: densitometric analysis of each component of the AMPK/SIRT1/PGC1α/PhoxO3a/PPARα/UCP2 pathway at different weeks of age in the kidneys of SHRSR **Panel B:** same analysis in SHRSP; **Panel C:** densitometric analysis of phosphoeNOS/eNOS and of NfKb in SHRSR; **Panel D:** same analysis in SHRSP. *p < 0.05 vs 6 weeks of age; **p < 0.01 vs 6 weeks of age; Δ*p* < 0.0001 vs 6 weeks of age

**Figure 5:**
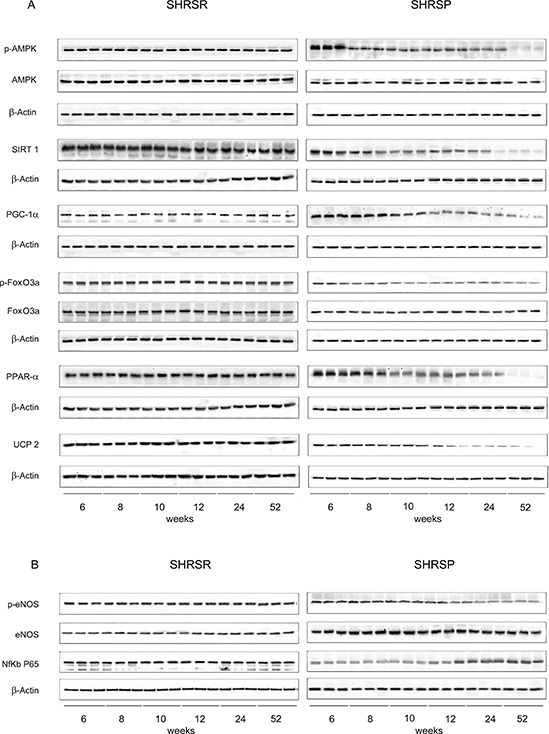
Panel A: western blots of AMPK/SIRT1/PGC1α/PhoxO3a/PPARα/UCP2 pathway throughout lifetime in the heart of SHRSR and SHRSP **Panel B:** western blots of phosphoeNOS/eNOS and of NfKb in the heart of the two strains.

1. AMPK/SIRT1/PGC1α/PhoxO3a/PPARα/UCP2. All components of UCP2 regulatory pathway and UCP2 itself were significantly down-regulated over lifetime in the brain, heart and kidneys of SHRSP but not in the same organs of SHRSR (Figures 1–6). Degree of protein down-regulation of all components of the pathway was already significant at 2 months of age.

**Figure 6:**
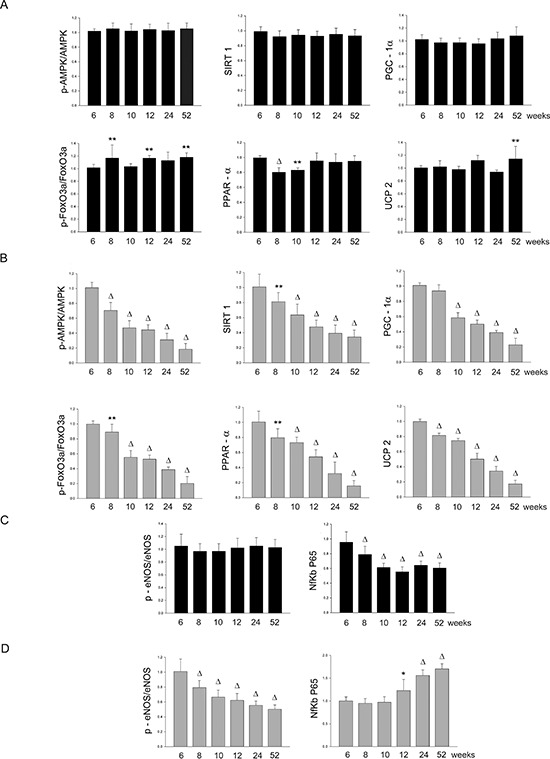
Panel A: densitometric analysis of each component of the AMPK/SIRT1/PGC1α/PhoxO3a/PPARα/UCP2 pathway at different weeks of age in the heart of SHRSR **Panel B:** same analysis in SHRSP; **Panel C:** densitometric analysis of phosphoeNOS/eNOS and of NfKb in SHRSR; **Panel D:** same analysis in SHRSP. *p < 0.05 vs 6 weeks of age; **p < 0.01 vs 6 weeks of age; Δ*p* < 0.0001 vs 6 weeks of age
2. Expression of eNOS was significantly decreased by 2 months of age in all tissues of SHRSP but not in SHRSR. In parallel, expression of Nf-kB increased progressively to become significant by 3 months of age in tissues of SHRSP only (Figures 1–6).
3. Analysis of carbonylated proteins revealed that this marker of tissue oxidative stress was significantly up-regulated, starting at 3 months of age, in tissues of SHRSP but not of SHRSR (Figures 7–9).

**Figure 7:**
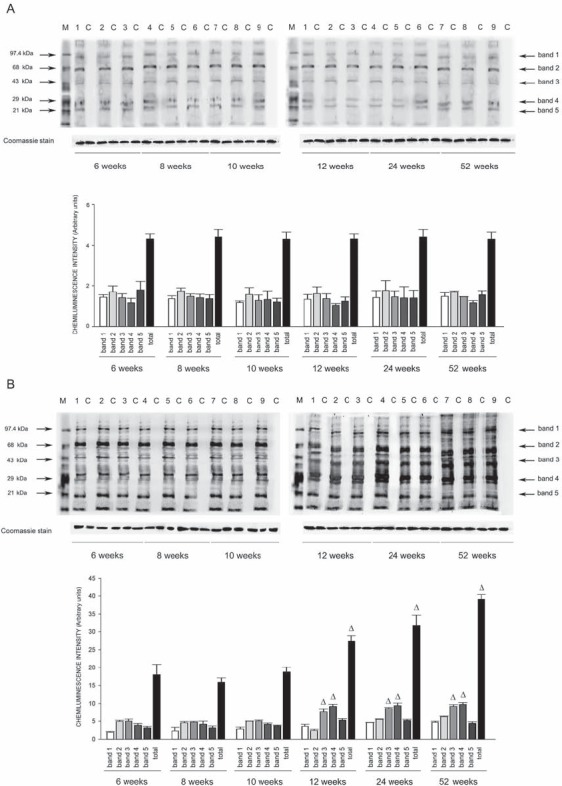
Western blots of intracellular protein extracts immunostained for carbonylated proteins using the Oxyblot Protein Oxidation Detection kit in the brains of SHRSR and SHRSP Each lane was loaded with 50 μg of total proteins. Lane M, DNP marker. Each sample is run with its own untreated control (C) Normalization for lane protein loading was performed using Coomassie staining. Bar graphs below the gel blots represent chemiluminenscence intensity relative to the gel loading band. Bands 1–5 refer to the most prominent bands on the blots (identified by arrows), whereas total refer to the total chemiluminescence intensity from all bands. Δ*p* < 0.0001 vs 6 weeks of age

**Figure 8:**
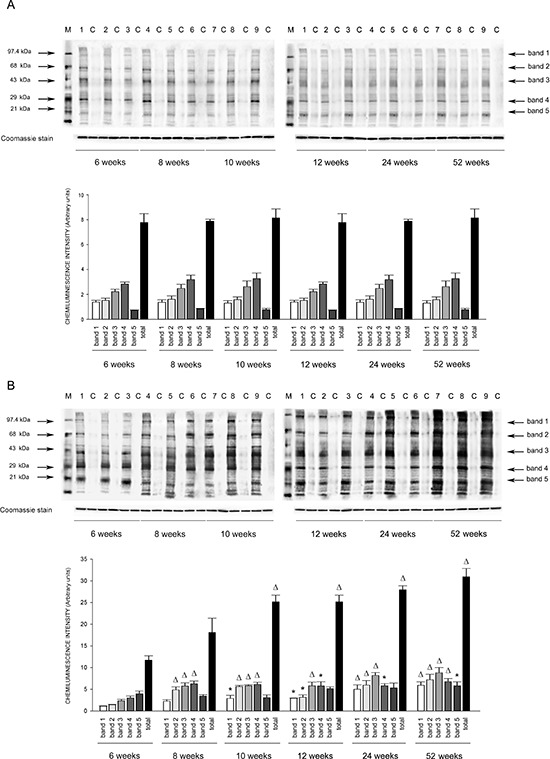
Western blots of intracellular protein extracts immunostained for carbonylated proteins using the Oxyblot Protein Oxidation Detection kit in the kidneys of SHRSR and SHRSP Each lane was loaded with 50 μg of total proteins. Lane M, DNP marker. Each sample is run with its own untreated control (C) Normalization for lane protein loading was performed using Coomassie staining. Bar graphs below the gel blots represent chemiluminenscence intensity relative to the gel loading band. Bands 1–5 refer to the most prominent bands on the blots (identified by arrows), whereas total refer to the total chemiluminescence intensity from all bands. *p < 0.05 vs 6 weeks of age; Δ*p* < 0.0001 vs 6 weeks of age

**Figure 9:**
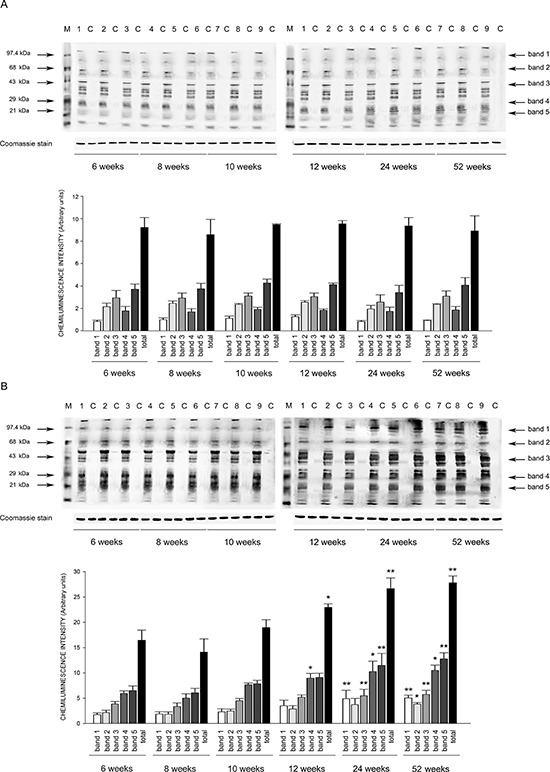
Western blots of intracellular protein extracts immunostained for carbonylated proteins using the Oxyblot Protein Oxidation Detection kit in the heart of SHRSR and SHRSP Each lane was loaded with 50 μg of total proteins. Lane M, DNP marker. Each sample is run with its own untreated control (C) Normalization for lane protein loading was performed using Coomassie staining. Bar graphs below the gel blots represent chemiluminenscence intensity relative to the gel loading band. Bands 1–5 refer to the most prominent bands on the blots (identified by arrows), whereas total refer to the total chemiluminescence intensity from all bands. *p < 0.05 vs 6 weeks of age; **p < 0.01 vs 6 weeks of age

### UCP2 gene expression in brain, kidneys and heart from SHRSP vs SHRSR

UCP2 gene expression decreased significantly in brain, heart and kidneys of SHRSP with aging. In particular, reduction of UCP2 mRNA levels in brain and heart tissues of SHRSP became significant at 10 weeks of age compared to levels detected at 6 weeks of age. In contrast, UCP2 mRNA levels increased significantly in tissues of SHRSR, starting at 10 weeks of age (Table 2).

**Table 2:** UCP2 expression levels in organs of SHRSP as compared to SHRSR at different weeks of age

SHRSR	6 weeks	8 weeks	10 weeks	12 weeks	24 weeks	one year
Brain Kidneys Heart	1 1 1	1.8 ± 0.01 0.9 ± 0.02 1.8 ± 0.1	1.8 ± 0.04^Φ^ 2.2 ± 0.3^Φ^ 1.4 ± 0.1^Φ^	1.9 ± 0.06^Φ^ 2.9 ± 0.2^Φ^ 3.3 ± 0.2^Φ^	2.1 ± 0.01^Φ^ 3.6 ± 0.08^Φ^ 5.3 ± 0.5^Φ^	2.1 ± 0.07^Φ^ 4.4 ± 0.2^Φ^ 6.7 ± 0.2^Φ^

SHRSR	6 weeks	8 weeks	10 weeks	12 weeks	24 weeks	one year
Brain Kidneys Heart	1 1 1	0.8 ± 0.01^♦^ 0.6 ± 0.02^*^^ω^ 0.9 ± 0.2	0.7 ± 0.02^♦^^ω^ 0.3 ± 0.03^♦^^#^ 0.03 ± 0.01^♦^^#^	0.5 ± 0.3^♦^^#^ 0.07 ± 0.01^♦^^#^ 0.03 ± 0.003^♦^^#^	0.5 ± 0.05^♦^^#^ 0.02 ± 0.003^♦^^#^ 0.03 ± 0.002^♦^^#^	0.02 ± 0.01^♦^^#^ 0.01 ± 0.001^♦^^#^ 0.03 ± 0.01^♦^^#^

* *p* < 0.05 SHRSP vs corresponding time of life of SHRSR

♦ *p* < 0.0001 SHRSP vs corresponding time of life of SHRSR.

Φ *p* < 0.0001, SHRSR at each indicated time of life vs its own 6 weeks of age

ω *p* < 0.02, SHRSP at indicated time of life vs its own 6 weeks of age

# *p* < 0.0001, SHRSP at indicated time of life vs its own 6 weeks of age.

### Histological examination in brain, kidneys and heart of SHRSP vs SHRSR

**Figure 10:**
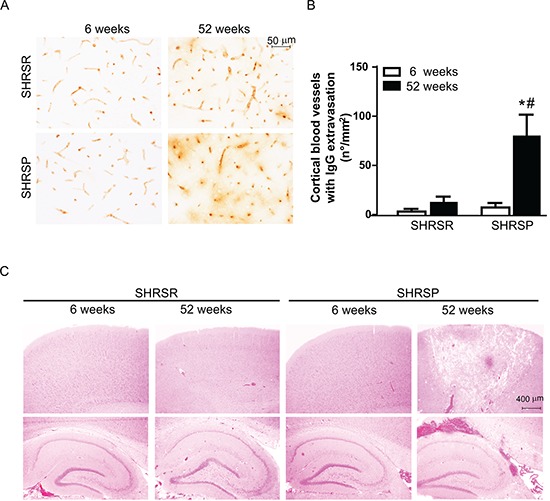
Panel A: representative images of IgG immunostaining detected in the frontal cortex of SHRSR and SHRSP **Panel B:** bar graphs represent quantification of number of cerebral blood vessels showing IgG extravasation density for mm^2^ in 20 randomly selected fields spanning the frontal cortex. *p < 0.05, one year-old SHRSP vs 6 week-old SHRSP; ^#^*p* < 0.05, one year-old SHRSP vs one year-old SHRSR. **Panel C:** H&E staining showing the presence of both ischemic and hemorrhagic lesions only in the brain of one year-old SHRSP.

We examined changes in blood brain barrier (BBB) permeability by assessing IgG extravasation. Substantial alterations in the BBB permeability were detected in SHRSP at one year of age, whereas no significant changes in IgG extravasation were found in age-matched SHRSR (Figure 10A, 10B). Consistently with these findings, histological analysis of H&E staining showed occurrence of both ischemic and haemorrhagic brain lesions only in SHRSP (Figure 10C).

**Figure 11:**
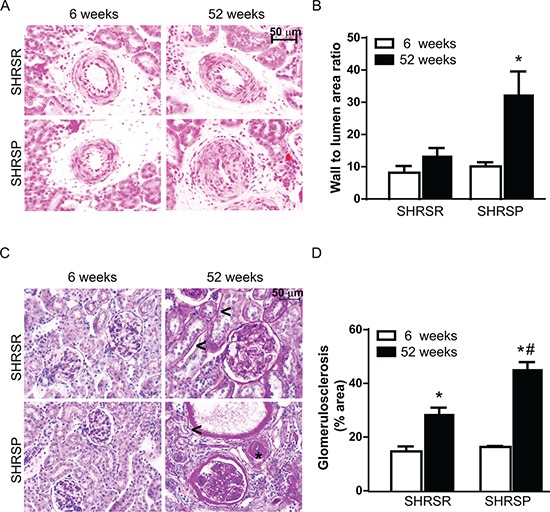
Panel A: representative images of renal arterioles of SHRSR and SHRSP at both 6 weeks and one year of age **Panel B:** bar graphs represent values of media-to-lumen area ratio. *p < 0.05, one year-old SHRSP vs 6 week-old SHRSP. **Panel C:** representative images of glomeruli from kidneys of SHRSR and SHRSP at both 6 weeks and one year of age. Presence of peritubular (<) and perivascular (*) fibrosis is indicated in one year-old rats. **Panel D:** bar graphs represent quantification of the glomerular percentage area positively stained with PAS. *p < 0.001, one year-old SHRSR vs 6 week-old SHRSR and one year-old SHRSP vs 6 week-old SHRSP; ^#^*p* < 0.001, one year-old SHRSP vs one year-old SHRSR.

**Figure 12:**
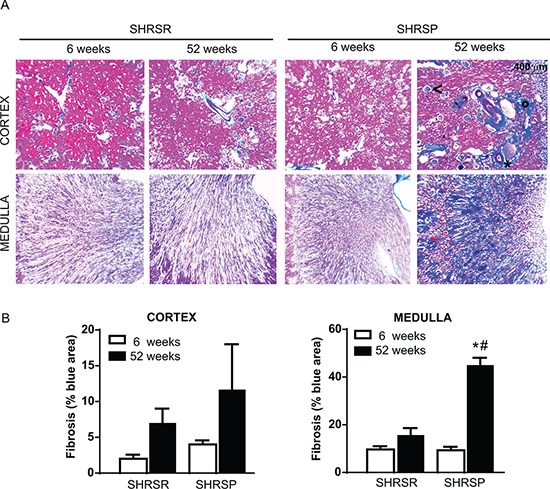
Panel A: representative images of renal fibrosis at both cortical and medullar level in SHRSR and SHRSP at both 6 weeks and one year of age Presence of interstitial (°), perivascular (*) and glomerular (<) fibrosis is indicated in the cortex of SHRSP rats at one year of age. **Panel B:** bar graphs represent quantification of fibrosis as percentage of blue area. *p < 0.001, one year-old SHRSP vs 6 week-old SHRSP; ^#^*p* < 0.001, one year-old SHRSP vs one year-old SHRSR.

Histopathological analysis of vessel remodelling in the kidneys showed a significant increase of medial thickening of renal arterioles of one year-old SHRSP as compared with both 6 week-old SHRSP and one year-old SHRSR (Figure 11A, 11B). No differences in vessel remodelling were found in SHRSR when comparing one year-old with 6 week-old rats (Figure 11A, 11B). Glomerulosclerosis was detected in both rat strains at one year of age, but it was more severe in SHRSP (Figure 11C, 11D). Trichrome staining showed a significant increase in medullar fibrosis in one year-old SHRSP as compared with both 6 week-old SHRSP and one year-old SHRSR. Again, no differences were found in the extent of interstitial fibrosis in SHRSR (Figure 12).

**Figure 13:**
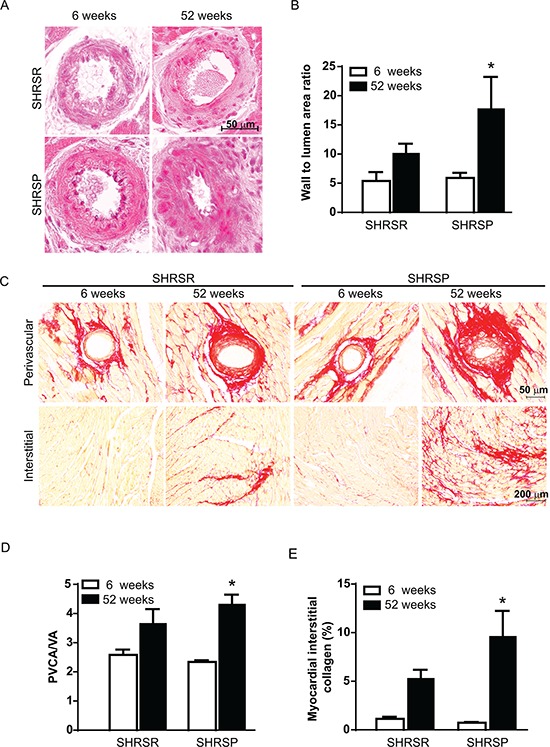
Panel A: representative images of intramyocardial coronary arterioles of SHRSR and SHRSP at 6 weeks and one year of age **Panel B:** bar graphs represent values of media-to-lumen area ratio. *p < 0.05, one year-old SHRSP vs 6 week-old SHRSP. **Panel C:** representative images of perivascular and myocardial interstitial collagen accumulation. **Panel D:** bar graphs represent perivascular collagen area-to-vessel area ratio (PVCA/LA). **Panel E:** bar graphs represent myocardial interstitial collagen percentage area. *P < 0.05, one year-old SHRSP vs 6 week-old SHRSP.

Finally, a significant vessel remodelling was detected in myocardial arterioles of one year-old SHRSP as compared to 6 week-old SHRSP, whereas no significant differences were identified in SHRSR (Figure 13A, 13B). Sirius red staining analysis showed significantly increased perivascular and interstitial fibrosis in the heart of one year-old SHRSP as compared to 6 week-old SHRSP, whereas no differences were detected in SHRSR (Figure 13C–13E).

## DISCUSSION

The present study demonstrates for the first time a remarkably different modulation of UCP2 and of all components of UCP2 upstream regulatory pathway along with hypertension development and aging in all target organs of two closely related spontaneously hypertensive rat strains. In fact, we observed a significant early (8 weeks of age) down-regulation of AMPK/SIRT1/PGC1α/PhoxO3a/PPARα/UCP2 protein expression that progressed throughout lifetime in the brain, kidneys and heart of SHRSP but not in its strictly related control strain, the SHRSR. UCP2 gene expression showed consistent changes in all tissues of the two rat strains. Accordingly, tissue inflammation and oxidative stress increased with aging only in the SHRSP strain. Furthermore, evidence of more marked renal, cerebral and cardiac damage was detected in older SHRSP as compared to SHRSR. Blood pressure levels were higher in SHRSP than SHRSR by 3 months of age and thereafter.

The SHRSP strain is a unique and widely used animal model with higher predisposition to develop hypertensive cerebrovascular damage over lifetime as compared to SHRSR [13]. Its spontaneous predisposition to stroke, which is likely to result from elevated blood pressure levels, can be accelerated by feeding with a high sodium/low potassium diet [9–13]. Notably, occurrence of stroke is greater in high salt fed SHRSP despite SBP levels comparable to those of high salt fed SHRSR [10, 11]. Renal damage precedes cerebrovascular accidents in SHRSP, behaving as a predictive factor for stroke similarly to the human condition [14]. Several therapeutic interventions were shown to protect from target organ damage development in the SHRSP model [15–17].

Dissection of mechanisms underlying vascular and tissue lesions in SHRSP would help understanding the hypertensive human disease and its related target organ damage. Of note, existence of genetic factors directly contributing to both stroke and renal damage occurrence has been reported in this strain [10]. Interestingly, the gene encoding UCP2 maps nearby the lod score peak of a Quantitative Trait Locus for stroke (STR1) in SHRSP [10, 11].

Here we have confirmed that SHRSP develop a more severe form of essential hypertension with aging and show the consequent target organ damage particularly localized within brain and kidneys, consistently with a number of previous reports [9–13]. In particular, we found that a significantly increased proteinuria, as a marker of renal damage documented by glomerular and tubulo-interstitial fibrosis, preceded cerebrovascular disease development. Within the brain, evidence of BBB dysfunction associated with stroke occurrence, in agreement with previous findings [18, 19]. Moreover, we detected an increased degree of vascular remodelling, as well as of both perivascular and interstitial fibrosis, in the heart of older SHRSP as compared to age-matched SHRSR.

The novelty of our study relies on the observation that early significant reduction of mitochondrial uncoupling protein expression takes place and progresses with hypertension and organ injury development only in SHRSP. This might directly contribute to hypertensive vascular disease development in SHRSP because SHRSR, that are more resistant to both renal and cerebrovascular damage, maintained higher level of mitochondrial uncoupling throughout lifetime. Consistently, the differential uncoupling expression level detected in all examined organs of the two hypertensive rat strains was associated with different tissue levels of both oxidative stress and inflammation. The latter represent common molecular mechanisms underpinning vascular damage.

Although the mechanistic link between the AMPK/SIRT1/PGC1α/PhoxO3a/PPARα/UCP2 axis down-regulation and the severe hypertensive disease of SHRSP remains to be explored, it is worthwhile noticing that significant changes in UCP2 gene and protein expression in SHRSP vs SHRSR could be already detected at 8–10 weeks of age, when SBP levels were similar in the two strains. Therefore, based on our current evidence, the early AMPK/SIRT1/PGC1α/PhoxO3a/PPARα/UCP2 axis down-regulation, with yet undefined molecular basis, precedes the development of a severe hypertensive condition in SHRSP and it appears to be an independent phenomenon. It can not be excluded that UCP2 down-regulation, by contributing to renal damage and vascular remodelling and by sustaining vascular dysfunction, may contribute on its own to maintain higher blood pressure levels in SHRSP. Furthermore, the parallel eNOS down-regulation observed exclusively in SHRSP over lifetime, consistent with reduced PPARα expression [20], may also contribute, through decreased nitric oxide production, to promote both hypertension and vascular damage in SHRSP.

It is interesting to note that the data obtained through a lifetime monitoring resemble very closely those observed in kidneys of SHRSP compared to SHRSR after short time (4 weeks) exposure to high salt diet [11, 12]. In fact, renal UCP2, along with its upstream regulatory pathway, are markedly suppressed by high salt diet only in SHRSP, and this is independent of changes in BP [11, 12]. The same holds true, based on ongoing data of our laboratory, for UCP2 levels in the brain of high salt fed SHRSP (unpublished data). Therefore, the dietary regimen is able to unmask the spontaneous age- and hypertension-related predisposition to blunt expression of both UCP2 and its regulatory pathway, thus accelerating the higher susceptibility to vascular disease development of SHRSP (as a proper example of gene-environment interaction).

Consistent with our current data, AMPK phosphorylation, UCP2 upregulation and reduced ROS production restored the impaired cerebrovascular endothelium-dependent vasorelaxation of aged normotensive Sprague Dawley rats [21]. Furthermore, PPARs, that are nuclear transcription factors regulating the expression of many genes involved in lipid metabolism and energy homeostasis, appear to play a major role in the age-delaying effects of calorie restriction by modulating mitochondrial function and UCPs [22, 23]. On the other hand, FOXO, that are part of the UCP2 regulatory pathway, are a group of transcription factors involved in resistance to stress [24]. Thus, the UCP2 pathway appears to be involved in protection from vascular damage in the context of both hypertensive and normotensive conditions with advancing age.

Lifespan of SHRSP is known to be shorter than that of SHRSR [25]. It is well known that UCP2 activity physiologically contributes to the maintainance of redox balance by uncoupling mitochondrial respiration, promoting fatty acid oxidation and mitochondrial biogenesis and reducing ROS production. UCP2 might be considered as a key defensive protein able to positively influence biological aging by allowing the most efficient use of molecular oxygen. Through these mechanisms it may contribute to expand lifespan. In fact, conditions that promote longevity through enhanced aerobic metabolism, such as aerobic exercise, increase expression of UCP2 in tissues [26]. Increased mitochondrial uncoupling activity of different tissues predicts longer lifespan of rats compared with mice [27]. Mice with increased metabolic intensities and greater mitochondrial uncoupling show reduced ROS production and longer lifespan, as compared to mice with low metabolic intensities [28]. Similarly, adult flies with increased uncoupling protein activity by targeted expression of exogenous UCP have an extended lifespan [29, 30]. Based on all these findings, an “uncoupling-to-survive” hypothesis has been proposed stating that higher uncoupling leads to greater oxygen consumption, reduced mitochondrial membrane potential and reduced ROS generation [5, 31, 32]. In contrast, UCP2 deficiency enhances ROS generation and the resulting redox imbalance can damage multiple organs and tissues [33–36].

The current results obtained in SHRSP support the evidence that UCP2 reduction with aging has a negative influence on cell function, physiological function, and overall animal health. In fact, the remarkable decline in vascular antioxidant gene expression during aging and severe hypertension may accelerate vascular disease development, i.e. fibrinoid necrosis, and contribute to the shorter lifespan of the SHRSP by promoting cerebro and renal vascular pathology.

In summary, our current results, obtained from the comparison of two closely related spontaneously hypertensive rat strains over their lifetime, strenghten the hypothesis that UCP2 and mitochondrial uncoupling has a key role in the maintenance of cardiovascular health. Due to its wide expression throughout the body, UCP2 may represent an attractive target to preserve the cardiovascular system from both hypertension- and age-related damage as well as to extend lifespan.

## MATERIALS AND METHODS

### Animals

Male rats of both SHRSP and SHRSR strains (derived from the Berlin's colonies) were housed in the animal facility room of Neuromed Institution. Animals were kept at constant temperature with a 12 hrs day-night cycle and free access to regular rat chow and water ad libitum. At the age of 6, 8, 10, 12, 24 weeks and one year selected groups of rats (including 4 or 5 animals) from both strains were used for BW and SBP measurements. A 24 hrs urines collection was performed in metabolic cages. Thereafter, rats were sacrificed by cervical dislocation. At this time brain, heart and kidneys were removed for tissue proteins and total RNA extraction, as well as for histological analyses.

The study was approved by our Institutional review committee. The procedures involving animals and their care were carried out in accordance with our institutions guidelines, which comply with national and international laws and policies.

### Systolic blood pressure, body weight and proteinuria levels measurements

SBP was measured non invasively in conscious restrained rats by means of a tail-cuff sphygmomanometry (BP-2000 Blood Pressure Analysis System, Visitech Sistems Apex NC. USA). The SBP value given for each animal represented the mean of multiple measurements.

Protein concentrations were assessed in 24 hrs urine collections by the Bradford method, as previously reported [10].

### Total proteins extraction and western blotting

Total proteins were extracted from brain, heart and kidneys of both rat strains at each indicated time point. Tissues were weigthed and homogenized at 4°C in Triton-X lysis buffer (10 mM Tris-HCl, pH 7.4, 150 mM NaCl, 1% Triton X-100, 1 mM EDTA, 10% glycerol, 1 mM phenylmethylsulfonyl fluoride, 10 μg/ml leupeptin, 10 μg/ml aprotinin, 1 mM sodium orthovanadate, 50 mM sodium fluoride, and 10 mM β-glycerophosphate, all reagents purchased from Sigma Aldrich). After 20 min of incubation on ice, samples were centrifuged at 12′000 rpm for 20 min at 4°C. The supernatants containing soluble proteins were collected. Protein concentrations were determined as specified above. Samples containing equal amounts of total proteins (50 μg) were separated on 12% SDS-PAGE and transferred to polyvinylidene difluoride membranes (Amersham, Piscataway, New Jersey, USA). Non specific binding sites were blocked with 5% non fat dried milk for 2 hrs at room temperature. Membranes were then incubated overnight at 4°C with each antibody, following previously described conditions [10].

β-actin was used as the housekeeping protein. β-actin primary antibody was purchased from Sigma Aldrich; NF-kB p65 primary antibody was purchased from SantaCruz (Santa Cruz, CA, USA). UCP2 primary antibody was purchased from Calbiochem (San Diego, CA, USA). AMPK, p-AMPK, SIRT1, PGC1α, FoxO3a, PPARα, eNOS primary antibodies were purchased from Cell Signalling (Danvers, MA, USA). As secondary antibodies, goat anti-rabbit or goat anti-mouse, purchased from Santa Cruz, were used. Signals were revealed with an enhanced chemiluminescence detection system (Luminata Crescendo, Millipore, Darmstadt, Germany) and the immunoreactivity of bands was visualized on a high-performance chemiluminescence apparatus (ChemiDoc MP System, Bio-Rad). Protein bands were scanned and quantified densitometrically. They were finally normalized using β-actin levels.

### UCP2 gene expression in tissues of SHRSP and SHRSR at different weeks of age

Brain, heart and kidneys of each strain were immediately frozen after removal and subsequently extracted for total RNA. The latter was isolated by RNAzol procedure with Trizol reagent (Invitrogen, Milan, Italy), subjected to purification by RNeasy kit (Qiagen, Hilden Germany) and to Dnase I treatment by RNase-free DNase (Qiagen, Hilden Germany), according to manufacturer's instructions. Two μg of total RNA were used for cDNA synthesis using Super Script VILO master mix (Invitrogen, Milan, Italy). UCP2 RNA transcripts were measured by real-time absolute quantitative PCR (QRT-PCR) by using predesigned TaqMan Gene Expression assay (Applied Biosystem, Milan, Italy). UCP2 TaqMan RT-PCR was performed in a final volume of 20 μL containing 10 μL of TaqMan Universal PCR Master Mix 2X; 1 μL of UCP2 TaqMan Gene expression assay 20X and 2 μL of purified cDNA. A parallel reaction was performed to amplify β-actin as housekeeping gene using β-actin TaqMan Gene Expression assay 20X. The amplification reactions were performed with ViiA7 System (Applied Biosystem, Milan, Italy) by applying the following amplification program: 50°C for 2 min, 95°C for 10 min followed by 95°C for 15 sec and 60°C for 60 sec for a total of 40 cycles. Each TaqMan assay was run in duplicate. Results were finally expressed as relative levels of each RNA comparing the different time points to the 6 weeks of age as the reference point.

### Protein oxidation detection in tissues of SHRSP and SHRSR at different weeks of age

Oxyblot Protein Oxidation Detection Kit (Millipore, Billerica, MA, USA) was used, according to manufacturer's instructions, for immunoblot detection of carbonyl groups in total proteins extracted, as reported above, from brain, heart and kidneys of both strains at different weeks of age. Normalization for lane protein loading was performed using Coomassie staining. The abundance of protein carbonylation was assessed by densitometry of each lane on a chemiluminescence detection system, as described above for western blotting. Chemiluminescence intensity of 5 prominent bands relative to the gel loading band was used to compare levels of carbonylated proteins among the different experimental groups.

### Histological examination of tissues of SHRSP and SHRSR

Brains, hearts and kidneys of SHRSR and SHRSP rats sacrificed at either 6 weeks or one year of age were dissected out, fixed in Carnoy reagent [ethanol (60%), acetic acid (10%), and chloroform (30%)] and embedded in paraffin. Ten micrometer sections were used for histological analyses.

#### a) Histological and immunohistochemical analysis of brain samples

Ten μm sections were stained with hematoxylin and eosin (H&E) for examination of ischemic and haemorrhagic lesions. Additional 10 μm sections were immunostained for IgG to assess cerebral IgG extravasation as an index of blood brain barrier breakdown. For this reason, deparaffinized sections were soaked in 3% hydrogen peroxide to block endogenous peroxidase activity and were incubated for 1 hr with a biotinylated anti-rat antibody (1:200; Vector Laboratories, Burlingame, CA). The immunoreaction was visualized with diaminobenzidine. The cerebral vessels showing IgG extravasation density (number for mm^2^) was quantified in 20 randomly selected fields spanning the frontal cortex.

#### b) Renal histology

Ten μm sections were stained with: H&E for examination of arteriolar remodelling; Azan Masson's trichrome for examination of cortical and medulla fibrosis; periodic acid Schiff (PAS) for assessment of glomerular sclerosis. For each analysis, two central longitudinal sections from each kidney were examined.

Wall-to-lumen area ratio, as an index of arteriolar thickening, was analyzed on sections stained with H&E. Renal arterioles ranging in diameter from 25 to 100 μm were selected for the analysis. An average of 10 renal arterioles was considered for each animal.

Tubulo-interstitial fibrosis examined with the Azan Masson's trichrome was identified as percentage of the area visualized with blue staining compared with background.

Glomerular sclerosis was assessed as percentage of the area of glomeruli positively stained with PAS. A total of 20 glomeruli was examined for each animal.

#### c) Heart histology

Ten μm sections were stained with H&E for examination of intramyocardial arteriolar thickness or with a collagen specific stain (Sirius red F3BA in aqueous saturated picric acid) for examination of both perivascular and interstitial fibrosis. For each analysis, two trasnsverse sections from each heart were examined. Wall-to-lumen area ratio, as an index of arteriolar thickening, was analyzed on sections stained with H&E. Intramyocardial arterioles ranging in diameter from 25 to 100 μm were selected for the analysis. An average of 18 intramyocardial arterioles was considered for each animal. Perivascular collagen was examined on sections stained with Sirius red. In order to normalize the area of perivascular collagen surrounding vessels with different sizes, perivascular collagen content was represented as the perivascular collagen area-to-vessel area ratio (PVCA/VA). Finally, interstitial fibrosis was assessed on sections stained with Sirius red as percentage of the area visualized with red staining compared with background. Perivascular collagen was specifically excluded from this analysis.

### Statistical analysis

BW, SBP, urinary protein levels, densitometric data of western blots, UCP2 RTPCR levels, histopathological data are provided as means + SD. Intergroup means were compared using one-way analysis of variance (ANOVA) or Kruskal-Wallis test followed by Bonferroni post-hoc test or Mann-Whitney test with Bonferroni's correction, respectively. Comparisons between two groups were performed using two-way ANOVA followed by Bonferroni's correction. SPSS statistical software (SPSS inc., Chicago, Illinois, USA, version 12.0) was used for statistical analysis. Statistical significance was stated at the *p* < 0.05 level.
